# Supplementary figures and images for: The relationship between ethylene-induced autophagy and reactive oxygen species in Arabidopsis root cells during the early stages of waterlogging stress
Source: PeerJ. 2023 May 26;11:e15404. doi: 10.7717/peerj.15404 (PMC10226478; doi:10.7717/peerj.15404)

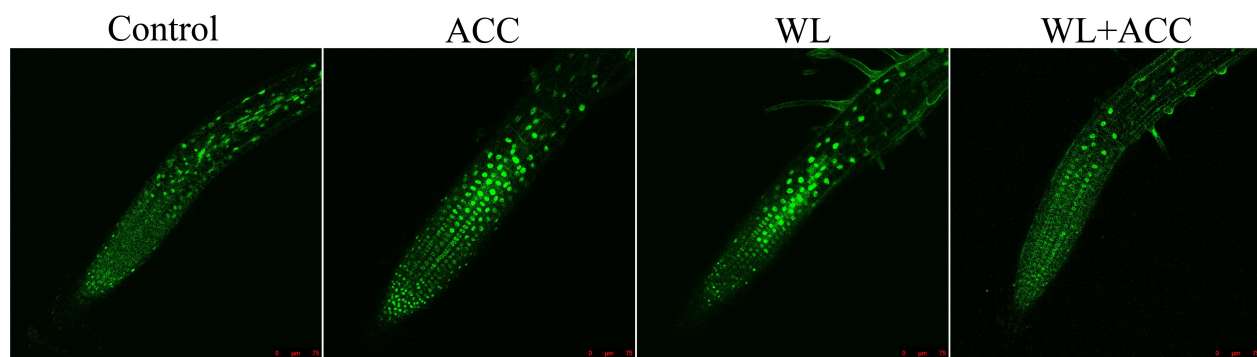

Supplement: Figure S1 — Seven-day-old seedlings of p35S::EIN3-GFP were observed using confocal microscopy at 12 h under different treatments, scale bar = 75 µm. All of the experiments were performed for three biological replications. [file peerj-11-15404-s001.pdf]

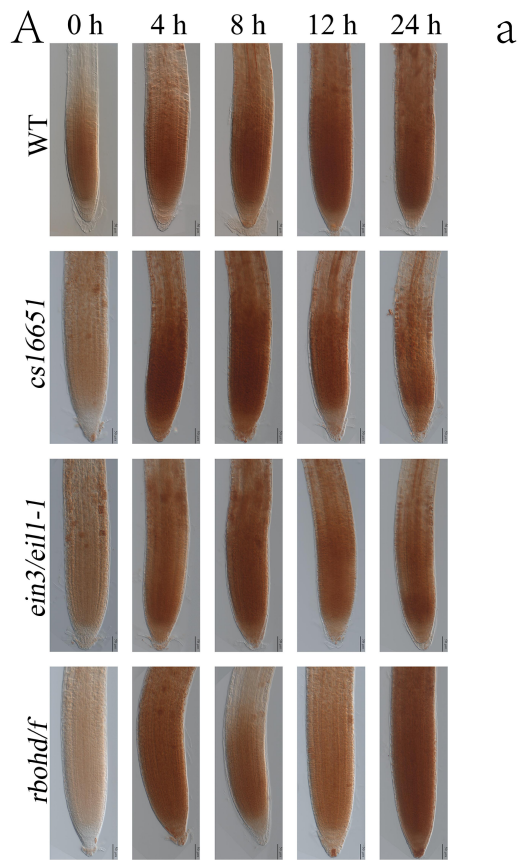

**a**

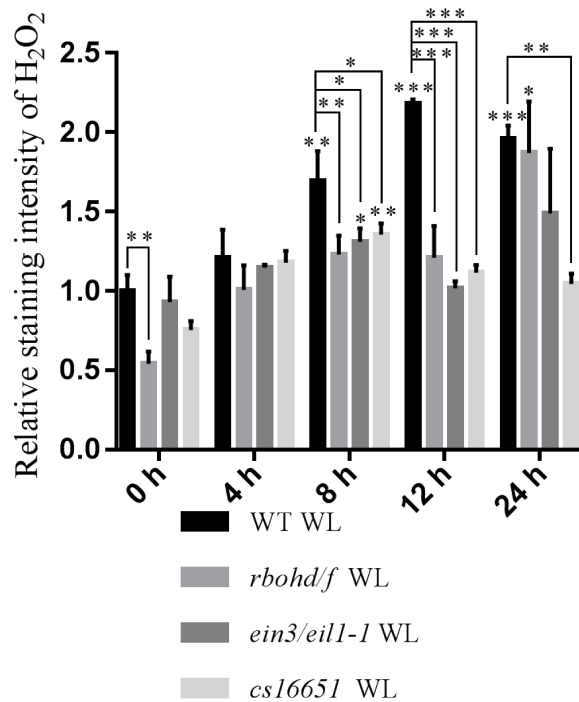

**B**

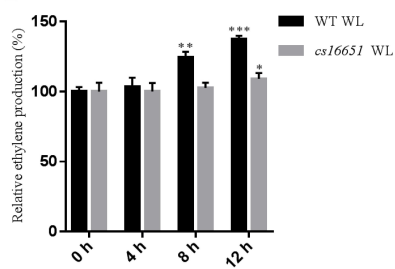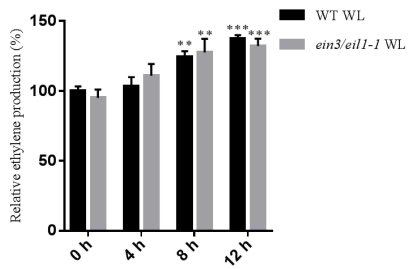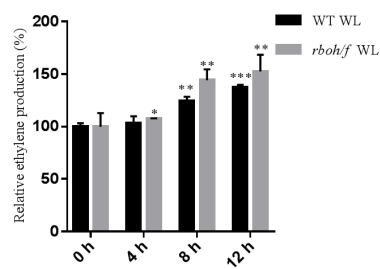

Supplement: Figure S2 — (A) DAB staining was used to detect changes in H2O2 levels in the roots of WT, ein3/eil1-1, cs16651, and rbohd/f seedlings under waterlogging treatment, with a scale bar of 50 µm. (A) The relative intensity was calculated based on the results in Fig. S2A. (B) The relative ethylene production (compared to WT at 0 h) was quantified for 7-day-old WT, ein3/eil1-1, cs16651, and rbohd/f seedlings under waterlogging treatment. All of the experiments were performed for three biological replications. Data shown are the mean ± SD (n = 3). * P < 0.05; ** P < 0.01; *** P < 0.001 by Student’s t-test. [file peerj-11-15404-s002.pdf]

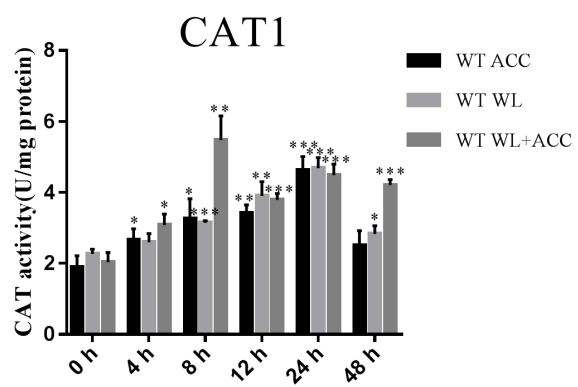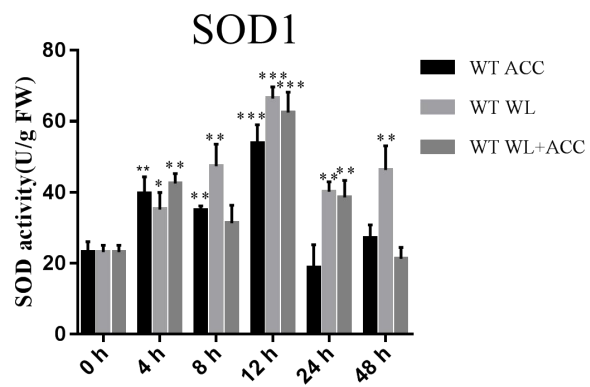

Supplement: Figure S3 — The activities of CAT andSOD enzymeswere measured in 7-day-old WT seedlings under different treatments for varying durations. All of the experiments were performed for three biological replications. Data shown are the mean ± SD (n = 3). * P < 0.05; ** P < 0.01; *** P < 0.001 by Student’s t-test. [file peerj-11-15404-s003.pdf]

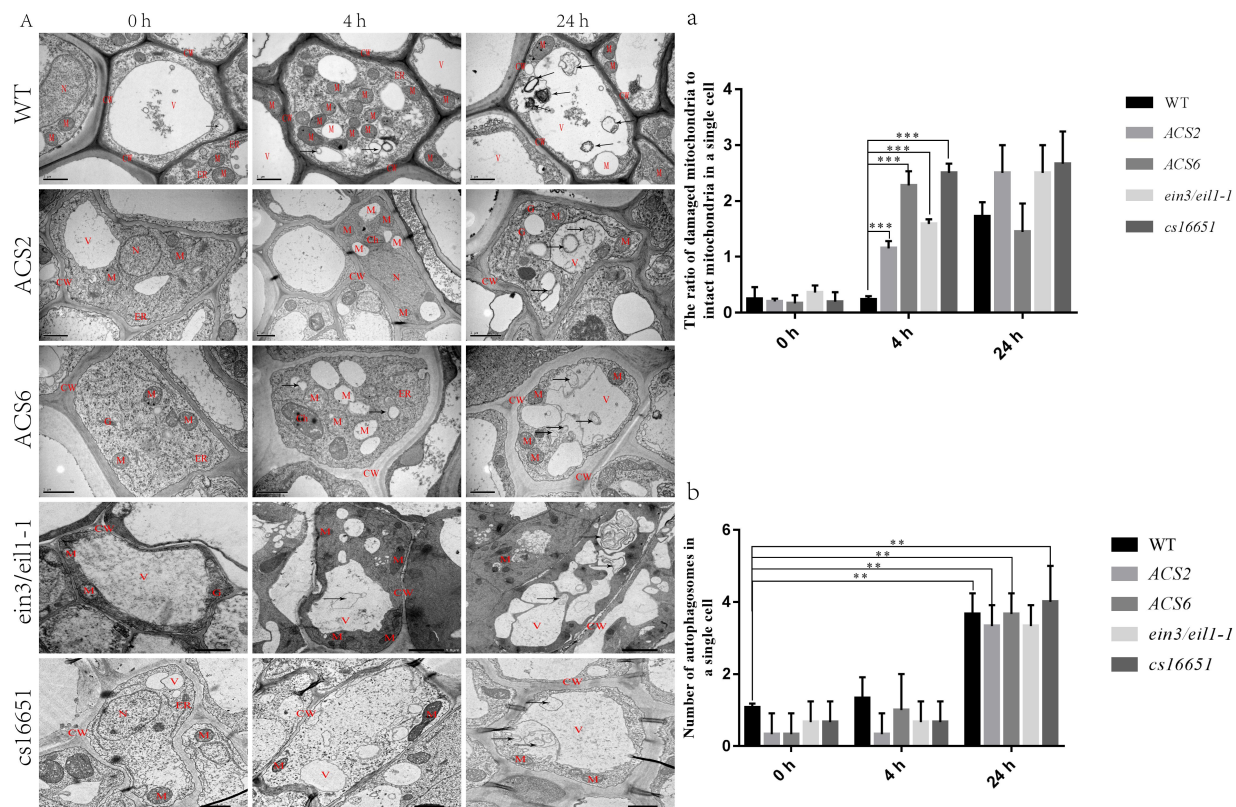

Supplement: Figure S4 — (A) The ultrastructure of roots from ethylene-related mutants ACS2, ACS6, ein3/eil1-1, and cs16651 seedlings was observed using transmission electron microscopy under waterlogging treatment at 0 h, 4 h, and 24 h. Arrows indicate autophagosomes or autophagic structures. CW denotes cell wall, M denotes mitochondrion or degraded mitochondria, ER denotes endoplasmic reticulum, G denotes Golgi, and V denotes vacuole. The scale bar is 1 µm. (A) The data are based on the ratio of damaged mitochondria to intact mitochondria in a single cell, as shown in Fig. S4A. (B) The data are based on the number of autophagosomes, as shown in Fig. S4A. All of the experiments were performed for three biological replications. Data shown are the mean ± SD (n = 3). * P < 0.05; ** P < 0.01; *** P < 0.001 by Student’s t-test. [file peerj-11-15404-s004.pdf]

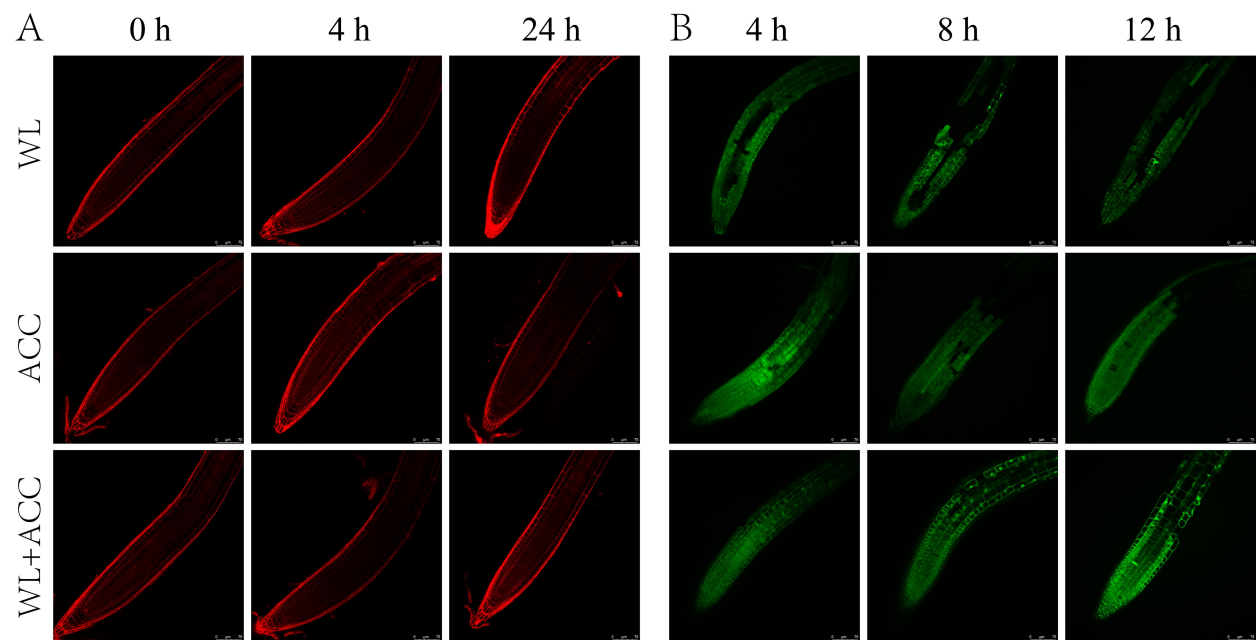

Supplement: Figure S5 — (A) and (B) show cell death and cell viability in root cells of WT observed by PI and FDA staining, respectively, under different treatments, with a scale bar of 75 µm. All of the experiments were performed for three biological replications. [file peerj-11-15404-s005.pdf]

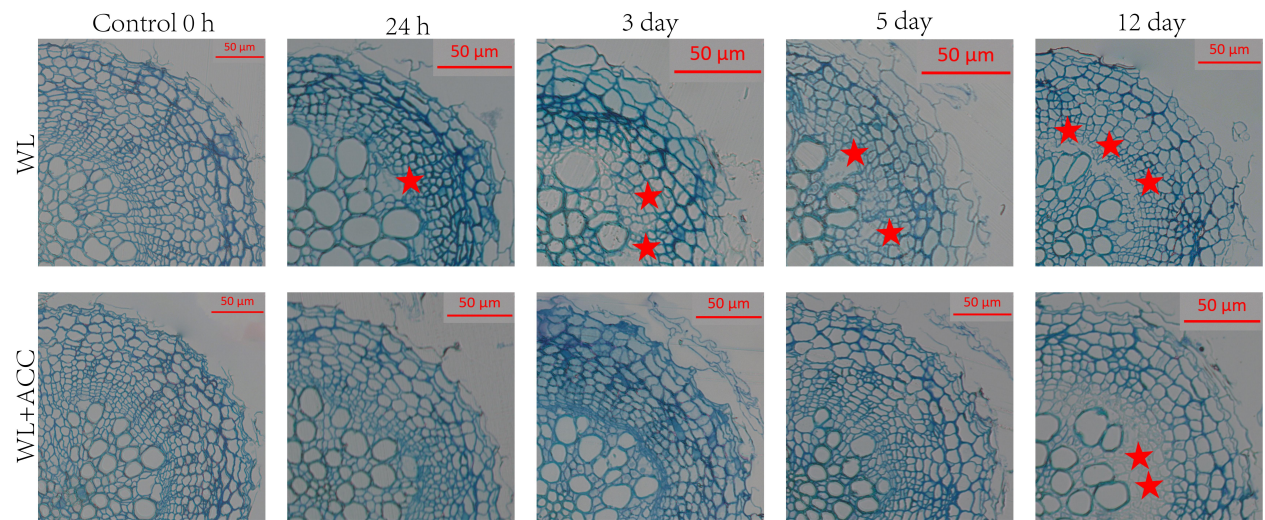

Supplement: Figure S6 — Seven-day-old WT seedlings were treated for varying durations, and the morphological changes in WT root cells were examined through semi-thin section experiments. The sections were then photographed using differential interference microscopy (Nikon 80i Eclipse), with the aerenchyma formed by the cells indicated by the red star area. The scale bar is 50 µm. All of the experiments were performed for three biological replications. [file peerj-11-15404-s006.pdf]

A

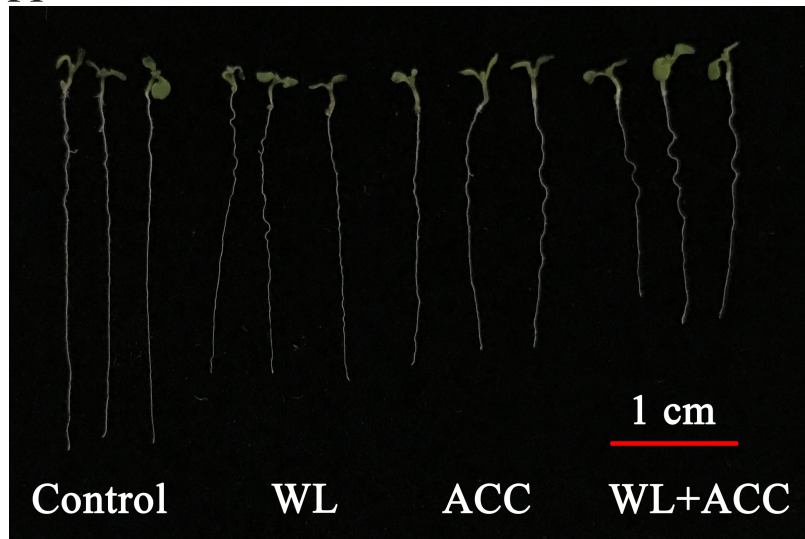

a

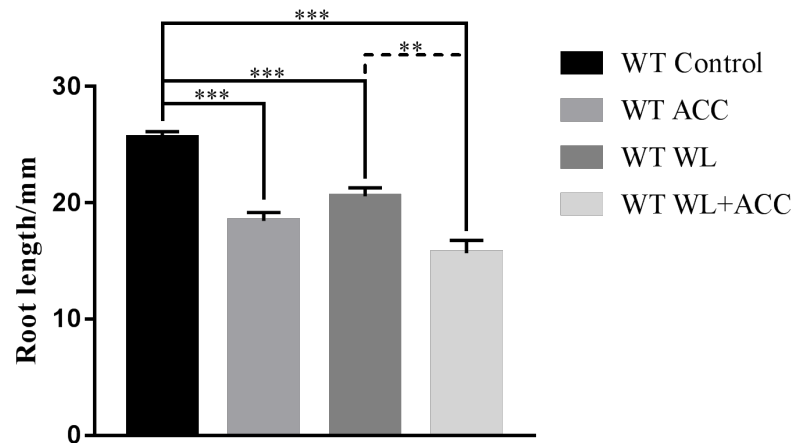

Supplement: Figure S7 — (A) Phenotypic changes in root length of 7-day-old WT seedlings under different treatments for 24 h, scale bar = 1 cm. (A) Statistical analysis of the root length in Fig. S7A. [file peerj-11-15404-s007.pdf]
