# Supplementary material for: The relationship between ethylene-induced autophagy and reactive oxygen species in Arabidopsis root cells during the early stages of waterlogging stress
Source: PeerJ. 2023 May 26;11:e15404. doi: 10.7717/peerj.15404 (PMC10226478; doi:10.7717/peerj.15404)
Supplement: Table S1 — Sequence of primers for several genes in RT-qPCR. [file peerj-11-15404-s008.docx]

**Table S1 Primer used for qRT-PCR**

| Gene | Forward primer | Reverse Primer |
| --- | --- | --- |
| *AtACTIN2* | TTGACTACGAGCAGGAGATGG | ACAAACGAGGGCTGGAACAAG |
| *ACO2* | TGGAACAAAAGGCCCAACCT | GAAGCTGGAGACCACTGACC |
| *ACS2* | TGTTTGACAAGCAACGCTGG | CGTCCATGTTGGCAAAGCAA |
| *ERF72* | CCGATTATGCCCCTCTCGTC | CACTGCCTCCTCTTTCACGT |
| *ERF73* | ATGGCGTCTTTGACTGCTCA | TCTGATGCTGAGCCTGAAGC |
| *EIN3* | TGCGTTGATGCAACACTGTG | GCAGTCAAAACGCCGACTTT |
| *ATG2* | AATGGATAGCAAGTGGAAGC | AGATAGACCTACCGTTAGCC |
| *ATG5* | ACTGATACCATGTGAAGGAG | GTATAGGCATCAAGATCACC |
| *ATG7* | GAAGATTGTCTAGGTCGTGG | CCTGCTTTCTCTTGTATCGG |
| *ATG8e* | CTGAAGCTGGAAGGATCAGG | GCTTGACATTAGCTCTCCTG |
| *ATG10* | ATCATACAAGGTTCCTGTGC | GATGTAGCTTGAACCATGGC |
| *SOD1* | ACAAGTTTGTACAAAAAAGCAGGCT | ACCACTTTGTACAAGAAAGCTGGGT |
| *CAT1* | AGGAGCCAATCACAGCC | TCAAGACCAAGCGACCA |
| *GST1* | AGGAGCCAATCACAGCC | GAAGATCGACCAAAGTGAAGTG |
